# Comparison of single-nucleotide variants identified by Illumina and Oxford Nanopore technologies in the context of a potential outbreak of Shiga toxin–producing *Escherichia coli*

**DOI:** 10.1093/gigascience/giz104

**Published:** 2019-08-21

**Authors:** David R Greig, Claire Jenkins, Saheer Gharbia, Timothy J Dallman

**Affiliations:** National Infection Service, Public Health England, 61 Colindale Avenue, London NW9 5EQ, UK

**Keywords:** Oxford Nanopore, Illumina, variant calling, STEC, outbreak

## Abstract

**Background:**

We aimed to compare Illumina and Oxford Nanopore Technology sequencing data from the 2 isolates of Shiga toxin–producing *Escherichia coli* (STEC) O157:H7 to determine whether concordant single-nucleotide variants were identified and whether inference of relatedness was consistent with the 2 technologies.

**Results:**

For the Illumina workflow, the time from DNA extraction to availability of results was ∼40 hours, whereas with the ONT workflow serotyping and Shiga toxin subtyping variant identification were available within 7 hours. After optimization of the ONT variant filtering, on average 95% of the discrepant positions between the technologies were accounted for by methylated positions found in the described 5-methylcytosine motif sequences, CC(A/T)GG. Of the few discrepant variants (6 and 7 difference for the 2 isolates) identified by the 2 technologies, it is likely that both methodologies contain false calls.

**Conclusions:**

Despite these discrepancies, Illumina and Oxford Nanopore Technology sequences from the same case were placed on the same phylogenetic location against a dense reference database of STEC O157:H7 genomes sequenced using the Illumina workflow. Robust single-nucleotide polymorphism typing using MinION-based variant calling is possible, and we provide evidence that the 2 technologies can be used interchangeably to type STEC O157:H7 in a public health setting.

## Background

Shiga toxin–producing *Escherichia coli* (STEC) O157:H7 is a zoonotic, foodborne pathogen defined by the presence of phage-encoded Shiga toxin genes (*stx*) [1]. Disease symptoms range from mild through to severe bloody diarrhoea, often accompanied by fever, abdominal cramps, and vomiting [2]. The infection can progress to haemolytic uraemic syndrome (HUS), characterized by kidney failure and/or cardiac and neurological complications [3, 4]. Transmission from an animal reservoir, mainly ruminants, occurs by direct contact with animals or their environment, or by the consumption of contaminated food products, with reported vehicles including beef and lamb meat, dairy products, raw vegetables, and salad [2, 4].

STEC O157:H7 belongs to multi-locus sequence type (MLST) clonal complex (CC) 11, with all but a small number of variants belonging to sequence type (ST) 11. CC11 comprises 3 main lineages (I, II, and I/II) and 7 sublineages (Ia, Ib, Ic, IIa, IIb, IIc, and I/II) [5]. There are 2 types of Shiga toxin, Stx1 and Stx2. Stx1 has 4 subtypes (1a–1d) and Stx2 has 7 subtypes (2a–2g). Subtypes 1a, 2a, 2c, and rarely 2d are found in STEC O157:H7. Strains harbouring *stx2a* are significantly associated with cases that develop HUS [2, 6]. As well as harbouring *stx* encoding prophage, STEC O157:H7 has an additional prophage repertoire accounting for ≥20% of the chromosome.

The implementation of whole-genome sequencing data for typing STEC has improved the detection and management of outbreaks of foodborne disease [6]. Single-nucleotide polymorphism (SNP) typing offers an unprecedented level of strain discrimination and can be used to quantify the genetic relatedness between groups of genomes. In general, for clonal bacteria, the fewer polymorphisms identified between pairs of strains, the less time since divergence from a common ancestor and therefore the increased likelihood that they are from the same source population. Therefore, it is paramount that variant detection for typing be accurate, highly specific, and concentrated on positions of neutral evolution to ensure the correct interpretation of the sequence data within the epidemiological context of an outbreak. It has been previously shown that different bioinformatics analysis approaches for variant identification exhibit detection variability [7, 8]. It is therefore important that, within a particular analysis, workflow parameters to filter identified variants to achieve optimum sensitivity and specificity are appropriately optimized.

Short-read sequencing platforms, such as those provided by Illumina, have been adopted by public health agencies for infectious disease surveillance worldwide [9] and have proved to be a robust and accurate method for quantifying relatedness between bacterial genomes. High-throughput Illumina sequencing, although cost-effective, often requires batch processing of hundreds of microbial isolates to achieve cost savings and therefore this approach offers less flexibility for urgent, small-scale sequencing often required during public health emergencies [10]. In contrast, Oxford Nanopore Technologies (ONT) offers a range of rapid real-time sequencing platforms from the portable MinION to the higher throughput GridION and PromethION models, although at this time lower read accuracy compared with Illumina data suggests that accurate variant calling may be problematic.

In September 2017, Public Health England (PHE) was notified of 2 cases of HUS in 2 children admitted to the same hospital on the same night. STEC O157:H7 was isolated from the faecal specimens of both cases. To rapidly determine whether the cases were part of a related phylogenetic cluster and therefore likely to be epidemiologically linked to each other or to any other cases in the PHE database, we sequenced both isolates using the MinION platform and integrated the ONT sequencing data with a dense reference database of Illumina sequences. We aimed to compare Illumina and ONT sequencing data from the 2 isolates to assess the utility of the ONT method for urgent, small-scale sequencing and to determine whether the same single-nucleotide variants were identified and whether inference of relatedness was consistent with the 2 technologies.

## Data Description

Paired-end FASTQ files were generated from the Illumina HiSeq 2500 for both samples (cases). Raw long-read data (FAST5) were generated from the MinION and base-called using Albacore (FASTQ) in real time. Both technologies' derived FASTQ reads were trimmed and filtered (Trimmomatic, Porechop, Filtlong) before being aligned (BWA, Minimap2) to a reference genome (NC_002695.1). Variant positions were called using GATK before being imported into SnapperDB. Full processing details can be found in the Methods section.

## Results

### Comparison of typing results generated by Illumina and ONT workflows

**Figure 1:**
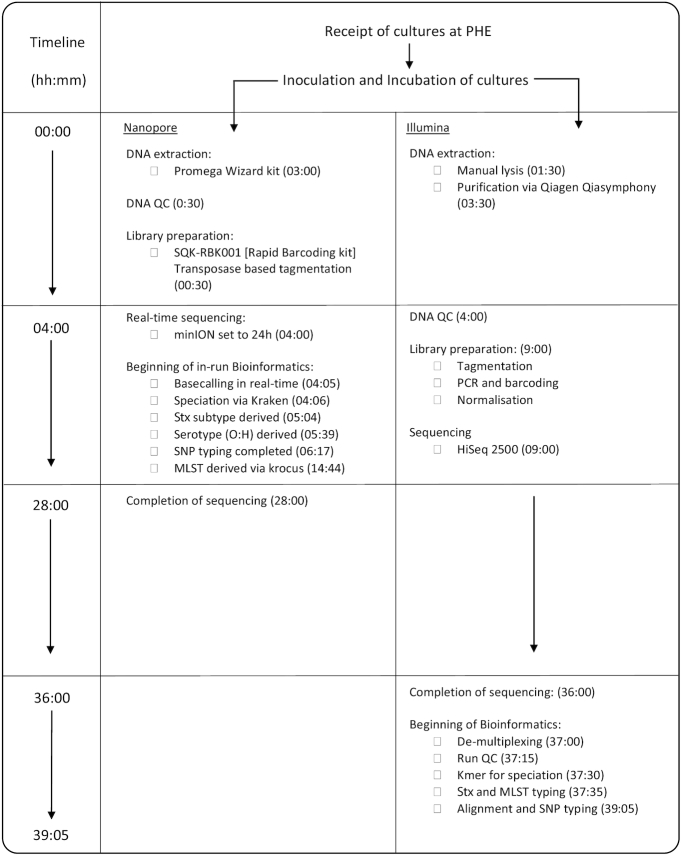
Figure showing comparative timeline from beginning DNA extraction to results generation for Oxford Nanopore and lllumina technologies. Times show the completion of the labelled event relative to the start of the assay (hh:mm). QC: quality control; Stx: Shiga toxin.

**Figure 2:**
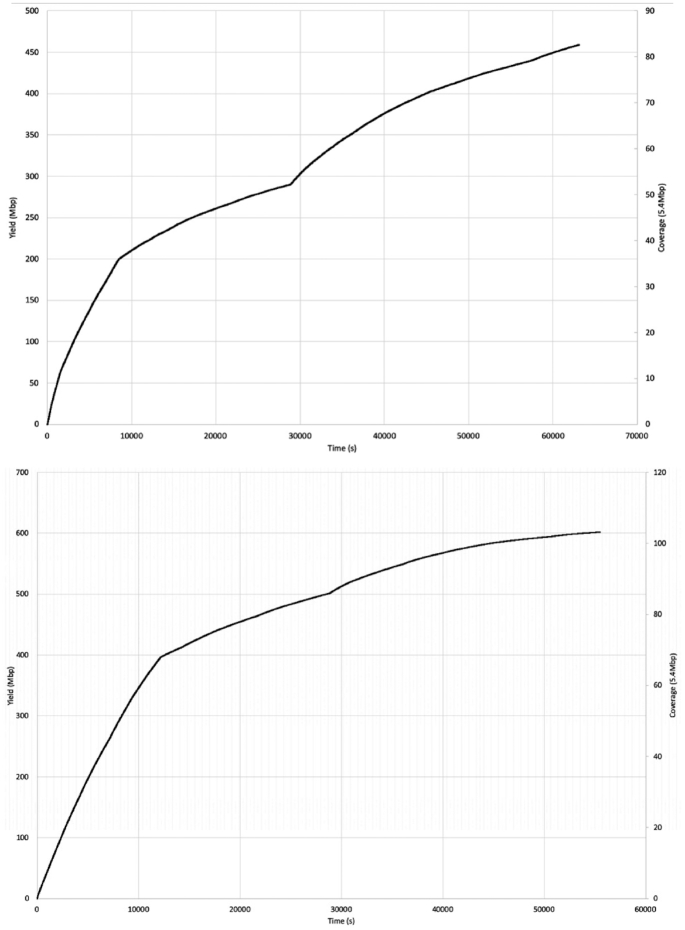
Time/yield/coverage graphs showing production of reads in real time and the associated cumulative mapping coverage. Case A is the graph on the top and Case B is below.

To consider the potential benefits of real-time sequencing to enhance opportunities for early outbreak detection, the timelines from DNA extraction to result generation for Illumina and ONT workflows were evaluated (Fig. 1) and the relationship between yield, time, and genome coverage plotted (Fig. 2). For the ONT workflow, the time from DNA extraction to completion of the sequencing run was 28 hours. A total yield of 0.45 Gb for the isolate from Case A and 0.59 Gb for the isolate from Case B was achieved, which corresponds to an equivalent coverage of the Sakai O157 STEC reference genome (5.4 Mb) of 81.29× and 108.30× for isolate A and B, respectively. The mean PHRED quality score for all reads in Case A was 9.87 and Case B was 9.47, which is ∼1 error every 10 bases. Base-calling and analysis was performed in real time, and serotyping, Shiga toxin subtyping, and variant identification were available within 6 hours and 20 minutes of the 24-hour sequencing run. With respect to the Illumina sequencing workflow, the time from DNA extraction to availability of results, assuming there were no breaks in the process, was just under 40 hours (Fig. 1).

The species identification, serotype, MLST profile, and Shiga toxin subtype results generated by both Illumina and ONT workflows were concordant with both isolates identified as *E. coli* O157:H7 ST11, *stx2a* and *stx2c*. During the ONT sequencing run, the bacterial species was unambiguously identified in <1 minute for both cases (Fig. 1). Additionally, using Krocus, a confirmed MLST was generated for Case A at 1:54 hours and Case B at 10:39 hours into the sequencing run. This was the point at which the last read required to generate a consensus on the MLST was base-called. By 93 minutes for Case A and 41 minutes for Case B, it was possible to determine the *E. coli* O157:H7 serotype, and *stx*2a and *stx*2c were detected at 58 and 24 minutes into the sequencing run for Case A and Case B, respectively.

### Optimization of ONT variant calling

**Figure 3:**
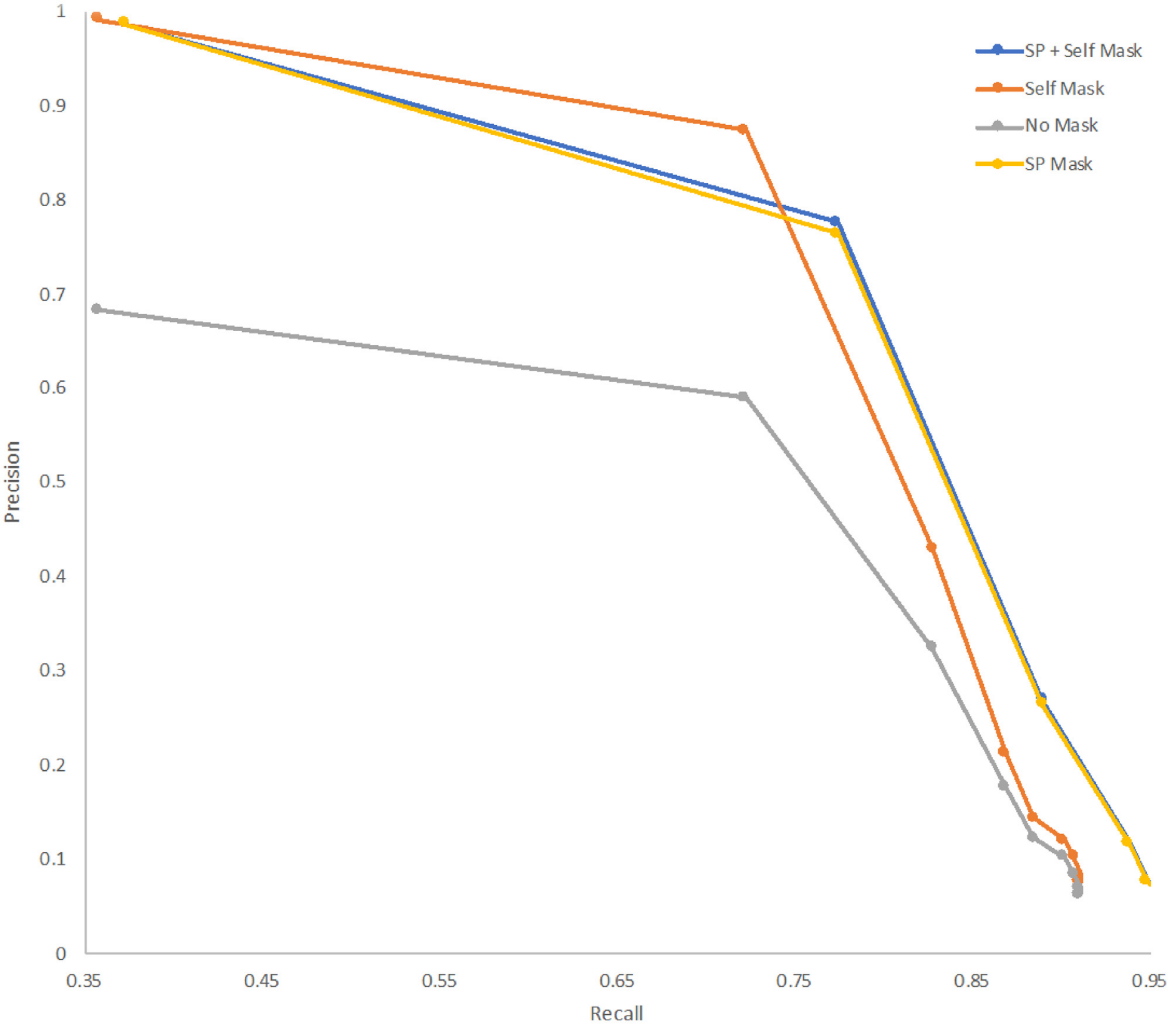
Precision vs recall of variant calling for an array of consensus ratio cut-offs and premasking strategies including masking positions annotated as “Sakai phage” ("SP") and positions that are ambiguously self-mapped (“Self”) with simulated Illumina FASTQs from the reference genome. Performed on case B.

**Figure 4:**
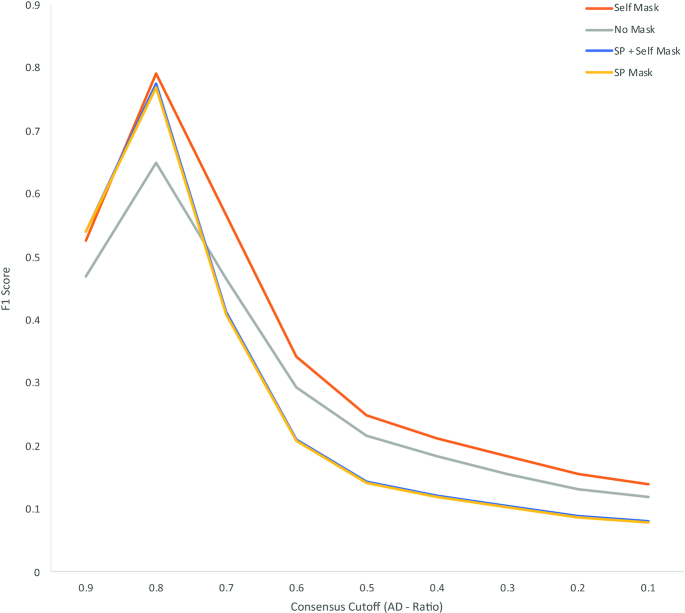
F1 Score for an array of consensus ratio cut-offs and premasking strategies including masking positions annotated as “Sakai phage” (“SP”) and positions that are ambiguously self-mapped (“Self”) with simulated Illumina FASTQs from the reference genome.

To compare Illumina and ONT sequences within a standardized framework it was necessary to optimize the parameters for variant filtering within GATK2 to compensate for the lower read accuracy observed in the ONT data. Using Case B for the optimization, base calls in the ONT data were classified as true-positive (variant base detected by both methods), false-positive (variant base in ONT, reference base in Illumina), true-negative (reference base in Illumina and ONT), or false-negative results (variant base in Illumina, reference base in ONT). To disregard areas of the genome that the ONT reads could map to (and therefore identify variants) but were ambiguously mapped with Illumina reads, prefiltering was performed by masking regions annotated as phage in the reference genome and those that could not be accurately self-mapped with simulated reference Illumina FASTQ reads. Fig. 3 plots the precision (the proportion of true-positive results with respect to all positive calls) against the recall/sensitivity (the proportion of true-positive results identified with respect to all true-positive results) for an array of consensus ratio cut-offs for each of the masking strategies. Similar areas under the curve (AUCs) were achieved for the different masking strategies, with slightly higher precision at lower recall achieved with “self-masking” (AUC, 0.71) and slightly higher recall at lower precision with explicit masking of the Sakai prophage (AUC, 0.75). The absence of a masking strategy markedly affects the precision of variant calling with ONT data, in comparison with Illumina as gold standard (AUC, 0.30). To identify the optimum consensus cut-off for filtering ONT variants processed through GATK the F1 score was calculated at each consensus cut-off. A consensus cut-off of 0.8 maximized the precision and recall (Fig. 4) irrespective of the filtering methods.

### Investigation of the discrepant variants identified between the Illumina and ONT data

After optimized quality and prophage filtering there were 266 and 101 base positions for Cases A and B, respectively, that were discordant between the ONT and Illumina sequencing data. The majority of discrepancies were where the ONT data identified a variant not identified in the Illumina data (261 of 266 [98.1%] and 95 of 101 [94.1%] discrepant base positions for Cases A and B, respectively). In contrast the Illumina data identified 5 (1.9%) discrepant base positions as variants for Case A and 6 (5.9%) for case B (Table 1) not identified by the ONT data.

**Table 1:** Breakdown of the total number of variants of each technology against the reference genome, followed by the numbers of masked variants within prophage or methylated positions

Variants and reason for omission	Case A		Case B	
**Total No. of variants against the reference genome after quality filtering**	2,076		1,424	
**Total No. of variants with masked due to location in phage**	708		531	
**Total No. of discrepant variants called between Case A and B alone**	266		101	
**Variants and reason for omission**	**Illumina VCF**	**ONT VCF**	**Illumina VCF**	**ONT VCF**
**No. of discrepant variants in each VCF**	5	261	6	95
**No. of discrepant variants with methylated positions masked**	0	260	0	94
**Final discrepant variants**	5	1	6	1

**Figure 5:**
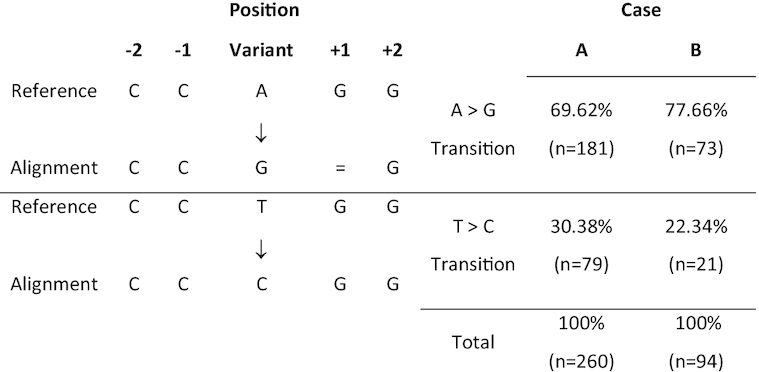
The 2 most common discrepancies in the ONT optimized GATK VCFs and a breakdown of the relative proportions of these transitions compared to the total number of discrepant SNPs for both cases.

For both cases the most common discrepant variants were adenines classified as guanines in the ONT data with respect to the Illumina data (and reference), accounting for 68.0% (181/266) for Case A and 72.3% (73/101) for Case B. The second most common discrepancy was thymine being classified as cytosine in the ONT data accounting for 29.7% (79/266) in Case A and 20.8% (21/101) in Case B (Table 1). Of the transitions described above, 97.7% (Case A) and 93.1% (Case B) occurred when the variant was between 2 homopolymeric regions of multiple cytosines and guanines (Fig. 5). These homopolymeric regions were similar to described DNA cytosine methylase (Dcm) binding sequences [11]. Nanopolish was subsequently used to identify likely Dcm, 5′-cytosine-phosphate-guanine-3′ (CpG), and DNA adenine methyltransferase (Dam) methylation sites in the ONT sequencing data and confirmed that 260 of 266 (97.7%) and 94 of 101 (93.1%) discrepant variants in the ONT data were classed as methylated for Cases A and B, respectively. All of these were determined to be Dcm methylation for both cases.

Once the methylated positions were masked from the analysis, there were a total of 6 (5 discrepant variants in Illumina and 1 ONT) and 7 (6 discrepant variants in Illumina and 1 ONT) discrepant SNPs between the ONT and Illumina data, for Cases A and B, respectively (Tables 2 and 3). Four discrepant Illumina variants are shared by both Case A and Case B. One shared variant was found in a non-coding region; another shared variant was found in *rhsC* encoding an RHS (rearrangement hotspot) protein defined by the presence of extended repeat regions. Two further shared variants were found in *dadX*, an alanine racemase gene. *dadX* is a paralogue of *alr*, also annotated as an alanine racemase in the *Sakai* reference genome with significant nucleotide similarity (>75% nucleotide identity). Both intra- and inter-gene repeats are known to be regions of potential false-positive calls with Illumina data due to mis-mapping. Of the 7 variants in the Illumina data found in either or both Case A and B, 5 were found to be homoplastic in the O157 population of 4,475 illumina sequences, arising independently, multiple times.

**Table 2:** Final discrepant SNPs between the Illumina data and ONT data for Case A

SNP	Position	Base in reference	Base in Illumina	Depth in Illumina	Base in ONT	Depth in ONT	Variant	Locus tag	Annotation
1	270,595	C	A	46	C	141	A	ECs0237	rhsC
2	379,516	A	G	114	A	100	G	NON CODING	N
3	1,681,338	C	G	59	C	61	G	ECs1685	Alanine racemase 2
4	1,681,339	G	C	57	G	61	C	ECs1685	Alanine racemase 2
5	2,636,513	T	C	91	T	69	C	ECs2674	Hypothetical protein
6	4,709,195	A	A	86	G	82	G	ECs4673	Membrane-bound ATP synthase epsilon-subunit AtpC

Also shown is the base as it is in the reference, the Ilumina called base and read depth at that position, and the same for the ONT data. Finally, also included is the locus tag relative to the reference genome and the gene annotation.

**Table 3:** Final discrepant SNPs between the Illumina data and ONT data for Case B

SNP	Position	Base in reference	Base in Illumina	Depth in Illumina	Base in ONT	Depth in ONT	Variant	Locus tag	Annotation
1	270,595	C	A	19	C	207	A	ECs0237	rhsC
2	379,516	A	G	52	A	124	G	NON CODING	N
3	1,681,338	C	G	44	C	86	G	ECs1685	Alanine racemase 2
4	1,681,339	G	C	41	G	86	C	ECs1685	Alanine racemase 2
5	2,033,176	T	G	34	T	85	G	ECs2049	Hypothetical protein
6	2,731,621	A	C	52	A	73	C	NON CODING	N
7	4,901,209	A	A	49	G	102	G	ECs4834	Superoxide dismutase SodA

Also shown is the base as it is in the reference, the Ilumina called base and read depth at that position, and the same for the ONT data. Finally, also included is the locus tag relative to the reference genome and the gene annotation.

### Phylogenetic analysis

**Figure 6:**
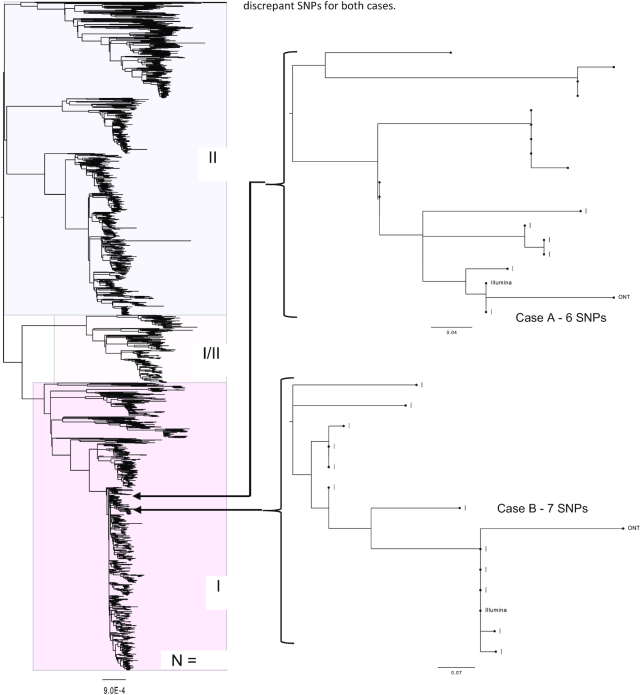
Maximum likelihood tree, of a “soft core” alignment of 4,475 genomes showing the tree lineages (I, I/II, and II) of STEC (Clonal Complex 11). Also showing where Oxford Nanopore and Illumina sequencing data are placed within the tree for each of the 2 cases. All methylated positions and prophage regions have been masked. Values represent the SNP differences between the Illumina and ONT data for both cases.

Using the optimized variant calling parameters both strains clustered phylogenetically in lineage Ic within a dense reference database of STEC O157:H7 genomes (n = 4,475). However, the genomes were located in distinct subclades (Fig. 6). It was, therefore, unlikely that the isolates originated from the same source, and it was concluded that Cases A and B were not epidemiologically linked. Following phylogenetic analysis of the Illumina SNP typing data (Fig. 6), Case A was designated a sporadic case. However, Case B clustered with a concurrent outbreak, already under investigation, comprising 3 additional cases. The Illumina sequence linked to Case B was zero SNPs different from the other 3 cases in the cluster, whereas the ONT sequence was 7 SNPs different, when excluding the methylated positions (Table 3). Based on the ONT sequencing data alone, this discrepancy would have led to uncertainty as to whether Case B was linked to the outbreak.

### Assembly profile

The ONT-only assembly resolved to 5 contigs (5.73 Mb) for Case A and 4 contigs (5.60 Mb) for Case B (Supplementary Table S1). In Case A, the 5 contigs were determined to be a single chromosomal contig, a single plasmid contig (pO157), and the 3 prophage duplications. In Case B, the 4 contigs were determined to be a single chromosomal contig with 2 plasmids (1 being the pO157). For Case A the assembly resolved to 25 contigs (5.51 Mb) with a hybrid assembly and 668 contigs (5.45 Mb) with an Illumina-only assembly. Case B resolved to 34 contigs (5.49 Mb) with a hybrid assembly and 575 contigs (5.42 mb) with an Illumina-only assembly.

Alignment of the assemblies (Supplementary Figs S1 and S2) revealed several locations within the ONT-only assembly that were absent in the hybrid and Illumina-only assemblies. In Case A, there were 8 regions only present within the ONT-only chromosome assembly, of which 7 are related to prophage regions (Supplementary Fig. S1). In case B, there were 10 chromosomal regions in the ONT-only assembly that did not align to the other assemblies. All 10 regions were associated with prophage regions (Supplementary Fig. S2).

## Discussion

In this study, the 2 isolates sequenced using ONT were unambiguously identified as STEC O157:H7 ST 11 *stx2a*/*stx2c* in <15 hours and it was possible to distinguish the genetic relatedness between the isolates within 377 minutes (i.e., 22 hours before the ONT sequencing run was scheduled to finish and just under 3 hours before the Illumina sequencing began). The whole-genome sequencing turnaround time, from DNA extraction and library preparation to sequencing and analysis via the Illumina workflow at PHE, is 3–6 days. Although this turnaround time is rapid for a service using batch processing on the HiSeq platforms, the sequencing approach using the MinION, whereby individual samples or small barcoded batches are loaded and results generated and analysed in real time, has the potential to be faster and more flexible. It should be noted that speed to result for ONT sequencing will be related to the amount of isolates run on a single flow cell because DNA molecules from different samples will compete to traverse a finite number of pores. This approach is therefore ideal for urgent, small-scale sequencing, often required during public health emergencies. In this scenario, analysis of the ONT data provided evidence that the 2 cases were not epidemiologically linked and, although efforts were made to determine the potential source of the infection for both cases through the National Enhanced STEC Surveillance System [2], an outbreak investigation was not initiated.

A current limitation of MinION sequencing is its lower read accuracy when compared to short-read technologies [12–16]. This accuracy has improved as the technology has matured but still falls short of the 99% accuracy offered by short-read platforms [15]. There are a number of factors that contribute to the current read accuracy in the nanopore data including structural similarity of nucleotides, simultaneous influence of multiple nucleotides on the signal, the non-uniform speed at which nucleotides pass through the pore, and the fact that the signal does not change within homopolymers [15].

Although analysis of the Illumina and ONT sequencing data placed the sequences on the same branch on the phylogeny, there were SNP discrepancies between the sequences generated by the 2 different workflows, even after optimization of the parameters. The vast majority of the discrepant SNPs (261/266 [98.1%] and 95/101 [94.1%] for Cases A and B, respectively) were attributed to variants identified in the ONT data and not the Illumina data. The majority of discrepancies (97.7% in Case A and 93.1% in Case B) were found in sequences that are the same as the known 5-methylcytosine motif sequences, CC(A/T)GG [11, 17] in the ONT data. Following a search of the ONT discrepant SNPs for CpG, Dam, and Dcm methylation using Nanopolish, the majority (97.7% and 93.1% for case A and B, respectively) of the ONT discrepant SNPs were identified in Dcm methylated regions.

As Nanopolish is detecting these methylated positions with the use of the raw FAST5 data, it is suggested that these particular discrepancies appear during the base-calling process. Albacore handles most methylation well across the 3 methylation models searched for by Nanopolish; e.g., only 94 of 13,504 methylated positions were considered incorrect by base calling for Case B. However, for mapping-based SNP typing, this level of error in base calling means that it is not possible to accurately determine the number of SNPs, thus potentially obscuring the true phylogenetic relationship between isolates of STEC O157:H7.

The optimization of variant filtering was performed using the Illumina data as a gold standard. However, it is possible that the alignment of the Illumina data might have generated false SNPs based on reads mapping to ambiguous regions of the genome, whereas the long reads obtained using the ONT workflow are able to resolve these ambiguous regions and call variants, or not, at these positions correctly. Because the Illumina data were used as the gold standard, in this scenario SNPs produced in the Illumina data would have been classed incorrectly as false-negative results in the ONT data. Discrepant variants identified in the Illumina data were attributed mainly to potentially false mapping of Illumina reads to homologous regions of the reference genome, variants that were misidentified at the same position independently in Cases A and B. Furthermore, comparison of assemblies generated by ONT reads, Illumina reads, and a hybrid approach highlights the extra genetic content accessible to ONT assemblies where variation can be quantified.

In this study an ONT sequencing workflow was used to rapidly rule out an epidemiological link between 2 children admitted to the same hospital on the same day with symptoms of HUS. The isolates of STEC O157:H7 from each child mapped to different clades within the same STEC O157:H7 lineage (Ic). We provide further evidence that SNP typing using MinION-based variant calling is possible when the coverage of the variation is high [15]. The error rate exhibited by ONT sequencing workflows continues to improve as a result of developments in the pore design, the library preparation methods, innovations in base-calling algorithms, and the introduction of post-sequencing correction tools, such as Nanopolish [15, 18]. Currently, both short- and long-read technologies are used for public health surveillance, and there is a need to integrate the outputs so that all the data can be analysed in the same way. Recently, Rang et al. [15] reiterated how the scientific community can make valuable contributions to improving ONT read accuracy by systematically comparing computational strategies as highlighted in this study and elsewhere [19]. Ongoing updates to the chemistry and software tools will facilitate the robust detection of SNPs, enabling ONT to compete with short-read platforms, ultimately enabling the 2 technologies to be used interchangeably in clinical and public health settings.

## Methods

### DNA extraction, library preparation, and Illumina sequencing

Genomic DNA (gDNA) was extracted from 2 strains of STEC O157 isolates from 2 HUS cases admitted to the same hospital on the same night. Using a Qiagen Qiasymphony (Qiagen, Hilden, Germany) in accordance with the manufacturer's instructions, gDNA was extracted and quantified using a Qubit and the BR dsDNA Assay Kit (Thermofisher Scientific, Waltham, MA, USA) to manufacturer's instructions. The sequencing library was prepared by fragmenting and tagging the purified gDNA using the Nextera XT DNA Sample Preparation Kits (Illumina, Cambridge, UK) to manufacturer's instructions. The prepared library was loaded onto an Illumina HiSeq 2500 (Illumina, Cambridge, UK) at PHE and sequencing perfomed in rapid run mode, yielding paired-end 100-bp reads.

### Processing and analysis of Illumina sequence data

FASTQ reads were processed using Trimmomatic v0.27 (Trimmomatic, RRID:SCR_011848) [20] to remove bases with a PHRED score of <30 from the leading and trailing ends, with reads <50 bp after quality trimming discarded. A *k*-mer approach [21] was used to confirm the species of the samples. ST assignment was performed using MOST v1.0 described by Tewolde et aal. [22]. *In silico* serotyping was performed by using GeneFinder [23], which uses Bowtie v2.2.5 (Bowtie, RRID:SCR_005476) [24] and Samtools v0.1.18 (SAMTOOLS, RRID:SCR_002105) [25] to align FASTQ reads to a multifasta containing the target genes (including *wzx, wzy*, and *fliC*). *Stx* subtyping was performed as described by Ashton et al. [26]. Illumina FASTQ reads were mapped to the Sakai STEC O157 reference genome (NC_002695.1) using BWA MEM v0.7.13 (BWA, RRID:SCR_010910) [27]. Variant positions were identified by GATK v2.6.5 UnifiedGenotyper (GATK, RRID:SCR_001876) [28] that passed the following parameters: >90% consensus, minimum read depth of 10, Mapping Quality (MQ) ≥ 30. Any variants called at positions that were within the known prophages in Sakai were masked from further analyses. The remaining variants were imported into SnapperDB v0.2.5 [29].

### DNA extraction, library preparation, and nanopore sequencing

To preserve DNA integrity for the nanopore sequencing, gDNA was extracted and purified using the Promega Wizard Genomic DNA Purification Kit (Promega, Madison, WI, USA) with minor alterations including doubled incubation times, no vigorous mixing steps (performed by inversion), and elution into 50 µL of double-processed nuclease-free water (Sigma-Aldrich, St. Louis, MO, USA). DNA was quantified using a Qubit and the HS (High Sensitivity) dsDNA Assay Kit (Thermofisher Scientific) according to the manufacturer's instructions. Library preparation was performed using the Rapid Barcoding Kit—SQK-RBK001 (ONT, Oxford, UK) with each sample's gDNA being barcoded by transposase-based tagmentation and pooled as per manufacturer's instructions. The prepared library was loaded on a FLO-MIN106 R9.4 flow cell (ONT, Oxford, UK) and sequenced using the MinION for 24 hours.

### Processing and analysis of nanopore sequence data

Raw FAST5 files were base-called and de-multiplexed in real time, as reads were being generated, using Albacore v2.1 (ONT) into FASTQ files. Run metrics were generated using Nanoplot v1.8.1 using default parameters [30]. Reads were processed through Porechop v0.2.1 using default parameters (R Wick, unpublished results) [31] to remove any barcodes and adaptors used in SQK-RBK001. For Case A 96,788 reads (10,214,353 bases) were adaptor trimmed and 386 (0.39%) chimeric reads split. For Case B 430,911 reads (34,888,999 bases) were adaptor trimmed and 513 (0.11%) chimeric reads split. Samples were speciated using Kraken v0.10.4 [32]. An MLST was assigned using Krocus with the following parameters: –kmer 15, –min_block_size 300, and –margin 500 [33]. S*tx* subtyping and serotyping was determined by aligning the base-called reads using minimap2 v2.2 [34] and Samtools v1.1 [25] to a multifasta containing the *Stx* and serotype encoding genes.

For reference-based variant calling FASTQ reads were mapped to the Sakai STEC O157 reference genome (NC_002695.1) using minimap2 v2.2 [34]. VCFs were produced using GATK v2.6.5 UnifiedGenotyper [28]. Any variants called at positions that were within the known prophages in Sakai were masked from further analyses. To determine the optimum consensus cut-off for ONT variant detection the VCF was filtered with sequentially decreasing ad-ratio values at 0.1 intervals. Using the Illumina variant calls as the gold standard, F1 scores (the weighted average of precision and recall) were calculated to determine the optimal ad-ratio for processing ONT data through GATK.

### Comparison of Illumina and Nanopore discrepant SNPs

Nanopolish [18] was also used to detect methylation across the ONT data to compare to the discrepant positions. This was performed using the call-methylation function, searching for 3 types of methylation including the DNA adenine methyltransferase (Dam), DNA cytosine methylase (Dcm), and 5′-cytosine-phosphate-guanine-3′ (CpG) models. The discrepant SNPs between the Illumina and ONT for both Case A and Case B were manually visualized in Tablet v1.17.08.17 [35] in order to elucidate the reason for the discrepancy. Discordant SNPs found within a homopolymeric region were also quantified.

### Generation of phylogenetic trees

Filtered VCF files for each of the Illumina and ONT sequencing data for each sample were incorporated into SnapperDB v0.2.5 [29] containing variant calls from 4,471 other STEC CC11 genomes generated through routine surveillance by PHE. SnapperDB v0.2.5 [29] was used to generate a whole-genome alignment of the 4,475 genomes (including both datasets for the selected strains for this study). Both methylated positions and prophage positions were masked from the alignment. The alignment was processed through Gubbins V2.0.0 [36] to account for recombination events. A maximum likelihood tree was then constructed using RAxML V8.1.17 [37].

### Assembly of ONT data

Trimmed ONT FASTQ files were assembled using Canu v1.6 (Canu, RRID:SCR_015880) [38]. Polishing of the assemblies was performed using Nanopolish v0.10.2 [18] using both the trimmed ONT FASTQs and FAST5s for each respective sample, accounting for methylation using the –methylation-aware option set to dcm. Assemblies were reoriented to start at the *dnaA* gene (NC_000913) from *E. coli* K12, using the fixstart parameter in circulator v1.5.5 [39].

### Hybrid assemblies

Trimmed ONT FASTQ files were assembled using Unicycler v0.4.2 [40] with the following parameters: min_fasta_length = 1000, mode = normal and -1 and -2 for the incorporation of each sample's equivalent Illumina FASTQ. Pilon v1.23 [41] was used to correct the assembly using the Illumina reads.

### Assembly of Illumina data

Illumina reads were assembled using SPAdes v3.13.0 (SPAdes, RRID:SCR_000131) [42] with the careful parameter activated and with *k-*mer lengths of 21, 33, 55, 65, 77, 83, and 91.

### Annotation

Prokka v1.13 [43] with the species set to E. coli was used to annotate the final assemblies.

Mauve snapshot_2015-02-25 (1) [44] using the “move contig” function was used to align each assembly to the ONT reference as they had the fewest contigs.

## Availability of supporting data and materials

The FASTQ files for the paired-read Illumina sequence data can be found on the NCBI SRA; Case A accession: SRR7184397, Case B accession: SRR6052929. The ONT FASTQ files, Case A accession: SRR7477814, Case B accession: SRR7477813. All files can be found under BioProject—PRJNA315192. The assemblies in the supplementary data were submitted to NCBI GenBank and are found under the following accessions: Nanopore assemblies (BioProject - PRJNA548323): ONT Case A: VIFR00000000, ONT Case B: VIFQ00000000. Illumina assemblies (BioProject - PRJNA315192): Illumina Case A: VIFT00000000, Illumina Case B: VIFS00000000. Hybrid assemblies (BioProject - PRJNA548322): Hybrid Case A: VIFP00000000, Hybrid Case B: VIFO00000000. All additional supporting data are available in the *GigaScience* GigaDB respository [45].

## Additional files

**Supplementary figure 1–**Mauve alignment showing regions of similarity between the ONT-only, hybrid, and Illumina-only assemblies (order descending) for Case A. Also showing the chromosomal regions in the ONT-only assembly that did not match the other assemblies (red arrows).

**Supplementary figure 2–**Mauve alignment showing regions of similarity between the ONT-only, hybrid, and Illumina-only assemblies (order descending) for Case B. Also showing the chromosomal regions in the ONT-only assembly that did not match the other assemblies (red arrows).

**Supplementary Table 1–**Number of contigs generated and size of assembly for each assembly method for both cases.

giz104_GIGA-D-19-00070_Original_Submission

giz104_GIGA-D-19-00070_Revision_1

giz104_Response_to_Reviewer_Comments_Original_Submission

giz104_Reviewer_1_Report_Original_Submission David Eccles -- 3/7/2019 Reviewed

giz104_Reviewer_2_Report_Original_Submission Oon Tek Ng -- 3/31/2019 Reviewed

giz104_Reviewer_3_Report_Original_Submission Brian Forde -- 4/2/2019 Reviewed

giz104_Supplemental_File

## Abbreviations

AUC: area under curve; bp: base pairs; BWA: Burrows-Wheeler aligner; CC: clonal complex; Dam: DNA adenine methyltransferase; Dcm: DNA cytosine methylase; GATK: Genome Analysis Toolkit; Gb: gigabase pairs; gDNA: genomic DNA; HUS: haemolytic uraemic syndrome; Mb: megabase pairs; MLST: multi-locus sequence type; NCBI: National Center for Biotechnology Information; ONT: Oxford Nanopore Technologies; PHE: Public Health England; RAxML: Randomized Axelerated Maximum Likelihood; SNP: single-nucleotide polymorphism; SPAdes: St. Petersburg genome assembler; SRA: Sequence Read Archive; ST: sequence type; STEC: Shiga toxin–producing *Escherichia coli*; VCF: variant call format.

## Competing interests

This project was part funded by Oxford Nanopore Technologies.

## Authors’ contributions

C.J. and T.J.D. conceptualized the project. C.J. and D.R.G. performed DNA extractions. D.R.G. performed library preparation and Nanopore sequencing. T.J.D. and D.R.G. processed Illumina sequence data. D.R.G. processed all ONT data. T.J.D. performed ONT optimization. D.R.G. performed methylation analysis. T.J.D. and D.R.G. performed Illumina and ONT data comparison. C.J. wrote the original draft. All authors performed manuscript editing.
