# Supplementary material for: Comparison of single-nucleotide variants identified by Illumina and Oxford Nanopore technologies in the context of a potential outbreak of Shiga toxin–producing Escherichia coli
Source: Gigascience. 2019 Aug 21;8(8):giz104. doi: 10.1093/gigascience/giz104 (PMC6703438; doi:10.1093/gigascience/giz104)
Supplement: giz104_Supplemental_File [file giz104_supplemental_file.docx]

**Supplementary Tables**

| **Case** | **# of contigs in ONT-only assembly (size bp)** | **# of contigs in hybrid assembly (size bp)** | **# of contigs in Illumina-only assembly (size bp)** |
| --- | --- | --- | --- |
| A | 5 (5,725,666 bp) | 25 (5,506,670 bp) | 668 (5,449,735 bp) |
| B | 4 (5,620,611 bp) | 34 (5,491,608 bp) | 575 (5,424,436 bp) |

**Table 1 –** Table showing the number of contigs generated and size of assembly for each assembly method for both cases.

**Supplementary Figures**


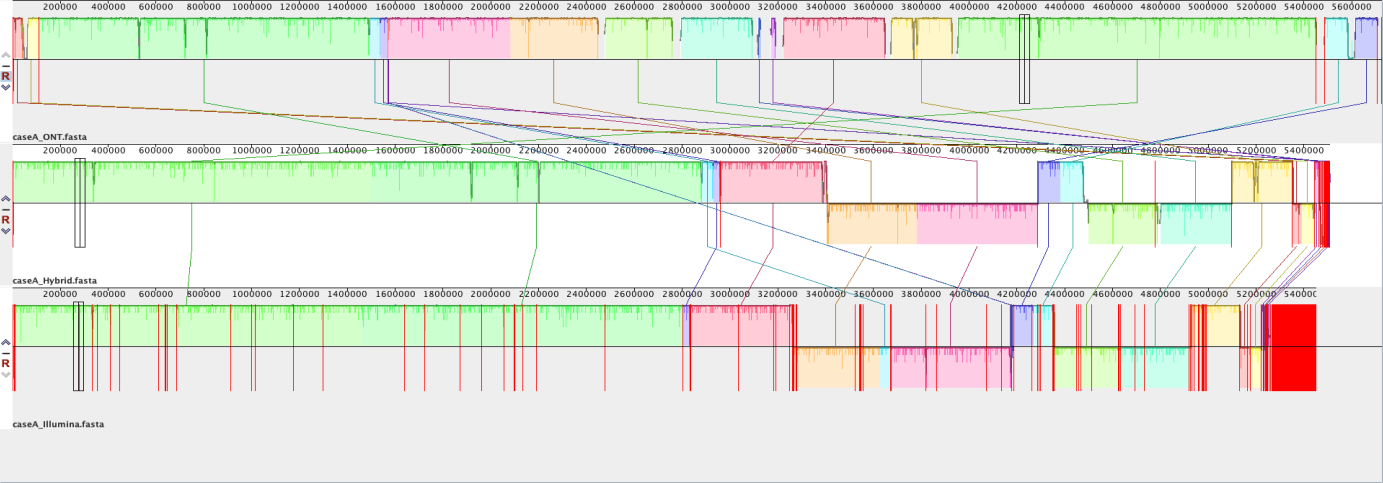


**Supplementary figure 1 –** Mauve alignment showing regions of similarity between the ONT-only, hybrid and Illumina-only assemblies (order descending) for Case A. Also showing the chromosomal regions in the ONT-only assembly that did not match the other assemblies (red arrows).


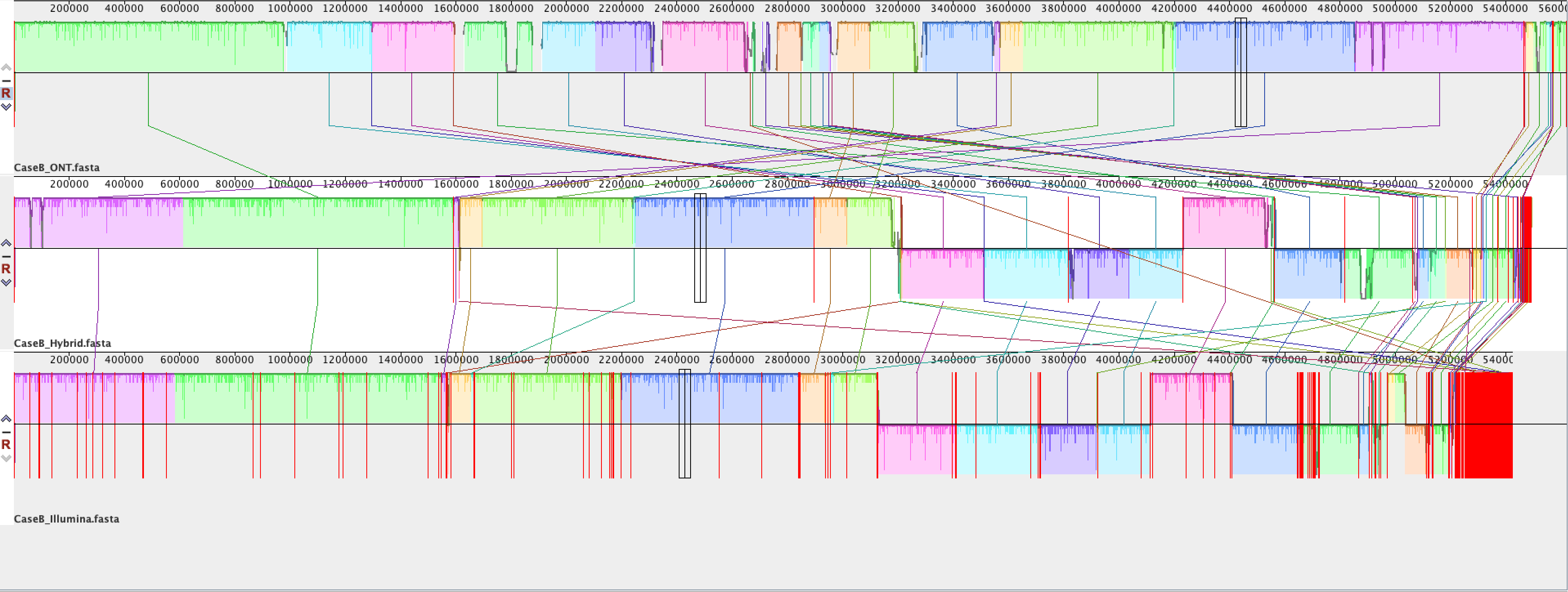


**Supplementary figure 2 –** Mauve alignment showing regions of similarity between the ONT-only, hybrid and Illumina-only assemblies (order descending) for Case B. Also showing the chromosomal regions in the ONT-only assembly that did not match the other assemblies (red arrows).
