# Supplementary material for: Comparison of single-nucleotide variants identified by Illumina and Oxford Nanopore technologies in the context of a potential outbreak of Shiga toxin–producing Escherichia coli
Source: Gigascience. 2019 Aug 21;8(8):giz104. doi: 10.1093/gigascience/giz104 (PMC6703438; doi:10.1093/gigascience/giz104)
Supplement: giz104_Response_to_Reviewer_Comments_Original_Submission [file giz104_response_to_reviewer_comments_original_submission.pdf]

Author's Response To Reviewer Comments

Close

Reviewer reports:

Reviewer #1: This manuscript describes a comparative analysis of Illumina and Nanopore sequencing, evaluating their usefulness for phylogenetic analysis, and for identifying genetic variants in outbreak situations.

The outcome of the research is somewhat surprising, given the expectation that Illumina sequencing represents the current gold-standard in sequencing accuracy. When eliminating systematic variation in base sequences caused by methylation, Nanopore sequencing appears to have similar accuracy to Illumina sequencing for the purpose of variant categorisation. When methylation is considered as a important feature, Nanopore sequencing demonstrates both a greater detection ability, and a faster turnaround time compared to Illumina sequencing. I am pleased to note that the supporting data was available at the time I carried out my review, and also pleased to be given the opportunity to approve this manuscript for publication.

I was specifically asked by the editors to state whether this represents "the state-of-the-art in terms of what this platform can do." It would be underselling the impact of these results to say no, that the current basecalling technology is better than what is presented in this paper. Due to the rapid advancement of nanopore sequencing technology in hardware, software, and chemistry, the yield and quality of results obtained from nanopore sequencing will be better than what is in *any* publication, even at the time when a
manuscript is submitted for review.

I recall seeing (and commenting) on David, Claire, Kathie, and Timothy's poster presented in April 2018, which seems to have been a similar (if not the same) study [https://twitter.com/gingerdavid92/status/987947325086666753]. This was the first study I'd seen that explicitly compared Illumina and Nanopore sequencing for phylogenetics [I accept there may be others that I haven't seen], and I'm pleased to see that they have incorporated an explicit analysis of methylation signals since then.

People have previously looked at phylogenetic trees for outbreak tracking with Nanopore sequencing (e.g. https://doi.org/10.2807%2F1560-7917.ES.2018.23.12.17-00140 [essentially cited in ref#10]), at accuracy estimates for Nanopore basecalling (e.g. https://doi.org/10.1101/543439), at hybrid isolate assembly from
barcoded Nanopore and Illumina reads (e.g. https://doi.org/10.1099%2Fmgen.0.000132 [cited]), and at comparing clinical turnaround time for Nanopore vs Illumina (e.g. https://doi.org/10.1128/JCM.02483-16), but this paper puts it all together into something that is still of substantial interest to the research community, as demonstrated by the social media impact of their preprint (https://doi.org/10.1101/570192).

In short, this manuscript is an excellent demonstration of what nanopore sequencing is capable of, represents the state-of-the-art (as I understand it) for public health investigations as presented in published papers, and I look forward to seeing more studies like this in the future.

Additional comments / questions:

1. Results, Tables 1/2 line 194-200 - Could you please either add in the legend that these SNPs were homoplasmic (very unlikely for ONT, somewhat possible for Illumina), or add the depth of the reference SNP bases to the table?

We have added the depth of sequencing for the final discrepant SNPs in to Table 2.
We have identified what SNPs in the were homoplasmic (5/7 Illumina variants) and a line in the text.

2. Methods, line 348 - These were barcoded reads that were processed through Porechop, which I understand can identify and filter out chimeric reads. Do you know how many reads were chimeric (we've typically observed <0.5% chimeric reads from rapid adapter preps, about 4% from ligation preps)?

We have added a line in the methods with these figures.

3. Discussion, line 239 - It is interesting to see from Figure 1 that all the nanopore data analysis was completed before the sequencing run had ended. Maybe this could be emphasised here: "within 377 minutes (i.e. over 20 hours *before* the sequencing run was scheduled to finish)."

We have added a short statement emphasising the difference between technologies.

4. Discussion, lines 250-259 - The final sentence doesn't seem to match the general idea of this paragraph. The paragraph is about single-base accuracy for single molecules (note: Illumina never sequences a single molecule to generate a base call), whereas the last sentence is about phylogenetics. I'd be happier if this paragraph were deleted entirely, as phylogenetics and error are also discussed in the next paragraph.

We have removed the last sentence from this paragraph. We think it is important to keep the current limitations to show what was state of the art at the time of this publication

5. Discusion, line 283 - "long reads... workflow is" -> "longreads... workflow are"

This has now been corrected.

6. Discussion, line 303 - "up-dates" -> "updates"

This has now been corrected.

7. Figure 1 - Why were different methods used for DNA extraction (i.e. Promega Wizard vs Manual lysis / Qiagen Qiasymphony)?

The Qiasymphony utilises a magnetic beads in beating protocol that causes DNA fragmentation leading to a decrease in high molecular weight DNA molecules and thereby sub-optimal for long read sequencing. Therefore, a commercial gDNA extraction kit with modifications to attempt to keep the DNA integrity as high as possible and thus generate longer reads. We have added a sentence in the text to reflect the motivation of DNA extraction method.

8. Figure 2 - The numbers are difficult to read. Could the axis text be made larger?

We have enlarged the text for the axis for both graphs in Figure 2.

9. Figure 4 - This should be a line graph (similar to figure 3). The points represent sampling of potential cutoff scores along a continuous distribution, and the score represents a single value rather than count data.

We have modified the figure accordingly

10. Figure 5 - Table 1 (Line 165-166) mentions that the total number of discrepant variants for case A and B is 266 and 101 respectively. This doesn't match the percentages and totals represented in Figure 5. I would expect that the Total line for A/B in Figure 5 should be 97.7% and 93% respectively, indicating that transitions comprised that proportion of the total variants. It would be useful to refer back to Tables 1 & 2 in the text for the other discrepant variants.

We have clarified this in the legend in figure 5. In table 1 we are showing the total number of discrepant positions within both Illumina and ONT vcfs, however in figure 5 we are discussing only the variant positions in the ONT data alone that were determined to be methylated. Figure 5 should equate to the row titled '# of discrepant variants with methylated positions masked' in Table 1.

11. Figure 6 - What do the numbers represent? It is not clear from the figure legend. These are presumably not bootstrap values, as they have a consistent ordering from top to bottom.

These values represent each respective case's SNP differences between the Illumina and ONT as demonstrated on the tree. We have updated the figure (6) legend to now include this clarification.

General questions:

Given that the Nanopore technology has improved in a number of different areas since this investigation was carried out (e.g. 9.4.1 Series D Flow cells, Field sequencing kit and/or RBK004, flip-flop basecaller), what (if anything) would be done differently if you had the opportunity to do this again?

Of your suggestions, the only one likely to make a significant difference would be re-basecalling with the most up to date flip-flop basecaller, we would hypothesise that this would reduce the number of SNP differences if it accounts for methylation better than previous basecalling algorithms. Another advancement is the new R10 pores which aims to improve the consensus accuracy is about 99.999% which again would reduce the number of total SNP differences but will most likely not account for methylation (unless a trained basecaller is also developed with these pores to account for methylation).

Are the assemblies available? I can't see anything about the assemblies in the "Availability" section.

We have now uploaded our assemblies to NCBI and have added a comment (with accession numbers) in the "Availability of supporting data" section (lines 409-416).

Reviewer #2: In their paper, Greig et al. compare the performance of Oxford Nanopore Technology (ONT) and Illumina sequencing in identifying, subtyping and classifying clinical isolates in the context of outbreak investigation. This study is of considerable interest to both research and medical communities in two key aspects. Firstly, it provides an in-depth assessment of the performance of ONT versus the current sequencing standard Illumina Technology, and identifies the main mechanistic reason for discrepancies between the technologies (DNA methylation), which would be of value in optimizing and improving Nanopore analysis workflows. Secondly, they demonstrate that their real-time ONT analysis pipeline is able to rapidly provide diagnostic calls (species identification, serotyping etc) with comparable accuracy to Illumina sequencing in a fraction of the time, which has major potential applications in outbreak investigation.

Given the utility of this study, I would be happy to recommend it for publication - please consider the following points to possibly improve it further.

1) Are the methods appropriate to the aims of the study, are they well described, and are necessary controls included?
1. The sample size of 2 isolates is small, but justified given the context of the study (2 urgent cases of children with HUS admitted to the same hospital on the same night). However, if the authors have any other isolates sequenced (in particular, a reference isolate closely related to the reference genome) that allow for the comparison of Illumina/ONT, they could include it as supplemental information to improve the robustness of their assessment.

Currently we have only processed these two STEC O157:H7 samples using this methodology, we are hoping to follow up this manuscript in the future on a larger set of samples.

2. Run parameters for bioinformatics tools are well optimized and described. It would also be of major help to the community if the authors are willing to share the code for their real-time analysis pipeline.

Each component/tool was run individually during this study. We have not developed an automated pipeline though this is something we plan to develop in the future.

3. The authors adequately discuss the limitations of ONT relative to Illumina sequencing with respect to their application in rapid diagnosis.
Fig 1/Methods - In the comparison of the ONT/Illumina workflows, we note that two different DNA extraction methods are used (manual + QiaSymphony cleanup for Illumina, Promega Wizard Genomic DNA Purification for ONT). Are the methods interchangeable for the purposes of the workflow?

See point 7 for reviewer 1

2) Are the conclusions adequately supported by the data shown?
Analyses are generally robust and well-supported, but we would like clarification on the following points:

4. Line 214 - When comparing the case B ONT sequence with the 3 concurrent outbreak isolates, was it compared against Illumina sequences, or Nanopore sequences? If the comparison was between ONT and Illumina sequences, the discordance might arise from differences in the base-calling/software methods, and might disappear if all isolates were sequenced with ONT and compared directly (or would the high error rate preclude a valid comparison?) Please clarify and comment.

The outbreak case B sequenced via ONT was compared to Illumina sequenced isolates (and the equivalent case B sample sequenced via Illumina). We believe that the observed differences are inherent errors in both technologies. Our hypothesis is in agreement with yours, that comparing ONT to ONT sequenced outbreak strains would remove these discrepancies.

5. Line 216-218 - Given that 7 SNPs is not too dramatic a difference one could still make the case that the cases are quite plausibly linked. Would you be able to set an approximate SNV threshold for concluding genetic linkage?

Currently we use 5 SNPs as a proxy to infer genetic linkage or sharing the same epidemiological source with Illumina sequenced strains, through extensive validation from sequencing known outbreaks. With ONT sequencing and due to the lack of background sequenced samples we are unable to comment on an “appropriate” SNP threshold. We would have to take into account comparing ONT to Illumina data, Illumina to Illumina data and ONT to ONT data to decide if each type of comparison requires a different threshold or if we could set a general one to cover all comparisons.

3) Please indicate the quality of language in the manuscript. Does it require a heavy editing for language and clarity?

6. The language of the manuscript is quite clear. Please correct "manufactures instructions" to "manufacturer's instructions".

This has now been corrected at each use.

7. I also feel that the title of the manuscript downplays the speed and relative accuracy of the ONT diagnostic pipeline - the focus of the title should not be on the comparison of SNVs, but rather the comparison of the overall performance of the two methods. A title reflecting this and highlighting the rapid, real-time analysis capability of ONT-based diagnostics would be able to better capture reader interest and increase the impact of the manuscript.

8. Similarly, the abstract should be edited to emphasize the speed and real-time analysis capability.

We feel that the current title is appropriate for this study as the emphasis was that in conjunction with the nanopore being a rapid, real-time portable sequencer the current dogma is that variant calling is currently out of scope for this technology due to the high error rate. This manuscript refutes that dogma.

4) Are you able to assess all statistics in the manuscript, including the appropriateness of statistical tests used?
Yes - our group has experience analysing similar datasets. The precision/recall analysis performed is straightforward and appropriate.

(This manuscript was co-reviewed with Weizhen Xu, a postdoctoral fellow in my research group)

Reviewer #3: The authors examine the feasibility of using Nanopore sequencing to characterise single nucleotide variants in clinically relevant outbreaks. The authors present an interesting and relevant comparison of sequencing technologies given the rapid uptake of WGS as a clinical diagnostic tool. The data set is clearly described and accession code for all data submitted to the SRA are available in the

manuscript and online. The methods are well described and all software/Bioinformatic tools are available online.

1. Although it can be addressed, my main concern with the manuscript is that the research was part funded by Oxford Nanopore. I believe the authors have not fully addressed the limitations of the Nanopore sequencing technology e.g. Cost, variability in throughput, etc. I? have highlighted some of these issues below. Platform limitations will also need to be included in the discussion

We have been more explicit to the funding received by Oxford Nanopore in the Acknowledgments. We have a paragraph on the limitations of ONT sequencing in terms of read accuracy and therefore variant detection which is the focus of this paper.

2. In the abstract and results the authors make a comparison of Illumina and ONT workflows of the time taken from DNA extraction to availability of results. The authors state that typing data (Shiga toxin subtyping and serotyping) was available within 7 hours while with Illumina it took ~40 hours to get these results. How do these time frames compare to standard laboratory based typing techniques that might be available in a diagnostic/pathology lab?

This paper covers the comparison between current methods deployed in the national reference laboratory – WGS by Illumina – with an alternative sequencing methodology ONT. It is out of scope of the paper to consider methods deployed in diagnostics e.g PCR

3. I would like to see a comparison of the sequencing costs for Illumina and Nanopore sequencing. A comparison against standard laboratory based typing techniques might also be beneficial to a broader audience (i leave that to the authors to decide).

Costing the sequencing technologies is not the focus of this paper and a would need to be a paper in its' own right considering labour / non-labour cost, deployment models etc. Also the value of such a comparison is incredibly time limited.

4. The authors state that the genetic relatedness of isolates could be determined at ~6 hours. I assume by genetic relatedness we are talking about variant/SNP typing. Can the authors explain how variants could be determined at 6 hours yet a MLST profile for Case B could not be determined until 10 hours had passed? Surely an inability to determine a MLST type indicates that the genome has not yet been sufficiently covered and it therefore unsuitable for variant typing.

Our SNP typing process requires an average genome coverage of 30x, the ONT sequencing took 6 hours to achieve this. As a result, as soon as 30x coverage is passed, this process can begin. Whereas, when sequencing the seven MLST genes, we are looking for enough coverage of those genes so that krocus can give us a confirmed result. This typically takes much longer, the last read was aligned to the seventh MLST gene at about 10 hours to then generate a full MLST result.

5. Additionally, in order to be cost effective multiple samples would need to sequenced on the same flowcell at the same time. Can the authors comment on what impacted multiplexing might have on the time frames described here?

This is correct, it is standard to multiplex several isolates per flow-cell. The higher the degree of multiplexing the increased pore competition we would expect the time to receive results per sample to increase. We have not performed this comparison. We have added this to the discussion

6. Can the authors comment as to why a number of regions (all describes as prophage with the exception of 1 in case A) were only present in the ONT-only chromosome assemblies? I find it odd that these regions were not present in the Illumina sequence data and are also absent from the hybrid assembly. can the authors comment on why this might be the case and what impact that might have for genome sequencing and assembly strategies moving forward?

The main reason for the smaller assemblies in the Illumina and hybrid approaches is the large amount of paralogous sequences in STEC O157 encoded on cryptic phage. These sequences (which are longer then the Illumina read length) collapse into a single contig or are broken up into many small contigs with only a single copy when in reality they are multi-copy in the genome. This results in smaller genomes when Illumina reads alone or as a hybrid.

7. With regards to the genome assemblies of case A and Case B can the authors provide information on the number of erroneous indels that were present in the Nanopore assemblies? I assume these errors were polished out but did the authors only use Nanopore sequence data or was Illumina data also required.?

To keep the comparison as true as possible we only polished the ONT assembly with ONT data using Nanopolish. It is difficult in this case to quantify how many of the indels associated in the ONT assembly are correct or conversely incorrect in the Illumina assembly as many fall in prophage regions.

8. Line 129: Remove the MLST allele numbers

This has now been corrected.

9. Line 385: form -> for(?)

This has now been corrected.

10. Table 1 is very hard to interrupt. Consider restructuring the table.

We have reformatted the table to make the breakdown of SNPs clearer.

Close
