# Supplementary material for: Comparison of single-nucleotide variants identified by Illumina and Oxford Nanopore technologies in the context of a potential outbreak of Shiga toxin–producing Escherichia coli
Source: Gigascience. 2019 Aug 21;8(8):giz104. doi: 10.1093/gigascience/giz104 (PMC6703438; doi:10.1093/gigascience/giz104)
Supplement: giz104_Reviewer_1_Report_Original_Submission — David Eccles -- 3/7/2019 Reviewed [file giz104_reviewer_1_report_original_submission.pdf]

Reviewer Report

Title: Comparison of single nucleotide variants identified by Illumina and Oxford Nanopore technologies in the context of a potential outbreak of Shiga Toxin Producing Escherichia coli

Version: Original Submission **Date:** 3/7/2019

Reviewer name: David Eccles

Reviewer Comments to Author:

This manuscript describes a comparative analysis of Illumina and Nanopore sequencing, evaluating their usefulness for phylogenetic analysis, and for identifying genetic variants in outbreak situations. The outcome of the research is somewhat surprising, given the expectation that Illumina sequencing represents the current gold-standard in sequencing accuracy. When eliminating systematic variation in base sequences caused by methylation, Nanopore sequencing appears to have similar accuracy to Illumina sequencing for the purpose of variant categorisation. When methylation is considered as a important feature, Nanopore sequencing demonstrates both a greater detection ability, and a faster turnaround time compared to Illumina sequencing. I am pleased to note that the supporting data was available at the time I carried out my review, and also pleased to be given the opportunity to approve this manuscript for publication.

--

I was specifically asked by the editors to state whether this represents "the state-of-the-art in terms of what this platform can do." It would be underselling the impact of these results to say no, that the current basecalling technology is better than what is presented in this paper. Due to the rapid advancement of nanopore sequencing technology in hardware, software, and chemistry, the yield and quality of results obtained from nanopore sequencing will be better than what is in *any* publication, even at the time when a manuscript is submitted for review.

I recall seeing (and commenting) on David, Claire, Kathie, and Timothy's poster presented in April 2018, which seems to have been a similar (if not the same) study [https://twitter.com/gingerdavid92/status/987947325086666753]. This was the first study I'd seen that explicitly compared Illumina and Nanopore sequencing for phylogenetics [I accept there may be others that I haven't seen], and I'm pleased to see that they have incorporated an explicit analysis of methylation signals since then.

People have previously looked at phylogenetic trees for outbreak
tracking with Nanopore sequencing
(e.g. https://doi.org/10.2807%2F1560-7917.ES.2018.23.12.17-00140 [essentially cited in ref#10]), at
accuracy estimates for Nanopore basecalling
(e.g. https://doi.org/10.1101/543439), at hybrid isolate assembly from
barcoded Nanopore and Illumina reads
(e.g. https://doi.org/10.1099%2Fmgen.0.000132 [cited]), and at comparing
clinical turnaround time for Nanopore vs Illumina
(e.g. https://doi.org/10.1128/JCM.02483-16), but this paper puts it
all together into something that is still of substantial interest to
the research community, as demonstrated by the social media impact of
their preprint (https://doi.org/10.1101/570192).
In short, this manuscript is an excellent demonstration of what
nanopore sequencing is capable of, represents the state-of-the-art (as
I understand it) for public health investigations as presented in
published papers, and I look forward to seeing more studies like this
in the future.
--
Additional comments / questions:
* Results, Tables 1/2 line 194-200 - Could you please either add in
the legend that these SNPs were homoplasmic (very unlikely for ONT,
somewhat possible for Illumina), or add the depth of the reference
SNP bases to the table?
* Methods, line 348 - These were barcoded reads that were processed
through Porechop, which I understand can identify and filter out
chimeric reads. Do you know how many reads were chimeric (we've
typically observed <0.5% chimeric reads from rapid adapter preps,
about 4% from ligation preps)?
* Discussion, line 239 - It is interesting to see from Figure 1 that
all the nanopore data analysis was completed before the sequencing
run had ended. Maybe this could be emphasised here:
"within 377 minutes (i.e. over 20 hours *before* the sequencing run
was scheduled to finish)."
* Discussion, lines 250-259 - The final sentence doesn't seem to match
the general idea of this paragraph. The paragraph is about
single-base accuracy for single molecules (note: Illumina never
sequences a single molecule to generate a base call), whereas the
last sentence is about phylogenetics. I'd be happier if this
paragraph were deleted entirely, as phylogenetics and error are also
discussed in the next paragraph.
* Discusion, line 283 -
"long reads... workflow is" -> "longreads... workflow are"

* Discussion, line 303 - "up-dates" -> "updates"
* Figure 1 - Why were different methods used for DNA extraction (i.e. Promega Wizard vs Manual lysis / Qiagen Qiasymphony)?
* Figure 2 - The numbers are difficult to read. Could the axis text be made larger?
* Figure 4 - This should be a line graph (similar to figure 3). The points represent sampling of potential cutoff scores along a continuous distribution, and the score represents a single value rather than count data.
* Figure 5 - Table 1 (Line 165-166) mentions that the total number of discrepant variants for case A and B is 266 and 101 respectively. This doesn't match the percentages and totals represented in Figure 5. I would expect that the Total line for A/B in Figure 5 should be 97.7% and 93% respectively, indicating that transitions comprised that proportion of the total variants. It would be useful to refer back to Tables 1 & 2 in the text for the other discrepant variants.
* Figure 6 - What do the numbers represent? It is not clear from the figure legend. These are presumably not bootstrap values, as they have a consistent ordering from top to bottom.

General questions:

* Given that the Nanopore technology has improved in a number of different areas since this investigation was carried out (e.g. 9.4.1 Series D Flow cells, Field sequencing kit and/or RBK004, flip-flop basecaller), what (if anything) would be done differently if you had the opportunity to do this again?
* Are the assemblies available? I can't see anything about the assemblies in the "Availability" section.

Methods

Are the methods appropriate to the aims of the study, are they well described, and are necessary controls included? Choose an item.

Conclusions

Are the conclusions adequately supported by the data shown? Choose an item.

Reporting Standards

Does the manuscript adhere to the journal's guidelines on [minimum standards of reporting?](#) Choose an item.

Choose an item.

Statistics

Are you able to assess all statistics in the manuscript, including the appropriateness of statistical tests used? Choose an item.

Quality of Written English

Please indicate the quality of language in the manuscript: Choose an item.

Declaration of Competing Interests

Please complete a declaration of competing interests, considering the following questions:

- Have you in the past five years received reimbursements, fees, funding, or salary from an organisation that may in any way gain or lose financially from the publication of this manuscript, either now or in the future?
- Do you hold any stocks or shares in an organisation that may in any way gain or lose financially from the publication of this manuscript, either now or in the future?
- Do you hold or are you currently applying for any patents relating to the content of the manuscript?
- Have you received reimbursements, fees, funding, or salary from an organization that holds or has applied for patents relating to the content of the manuscript?
- Do you have any other financial competing interests?
- Do you have any non-financial competing interests in relation to this paper?

If you can answer no to all of the above, write 'I declare that I have no competing interests' below. If your reply is yes to any, please give details below.

I have received workshop training, free kits, and flow cells from Oxford Nanopore Technologies in appreciation for my vocal work in supporting and helping others in the nanopore sequencing community.

I agree to the open peer review policy of the journal. I understand that my name will be included on my report to the authors and, if the manuscript is accepted for publication, my named report including any attachments I upload will be posted on the website along with the authors' responses. I agree for my report to be made available under an Open Access Creative Commons CC-BY license (http://creativecommons.org/licenses/by/4.0/). I understand that any comments which I do not wish to be included in my named report can be included as confidential comments to the editors, which will not be published.

I agree to the open peer review policy of the journal

To further support our reviewers, we have joined with Publons, where you can gain additional credit to further highlight your hard work (see: https://publons.com/journal/530/gigascience). On publication of

this paper, your review will be automatically added to Publons, you can then choose whether or not to claim your Publons credit. I understand this statement.

Yes Choose an item.
