# Supplementary material for: Comparison of single-nucleotide variants identified by Illumina and Oxford Nanopore technologies in the context of a potential outbreak of Shiga toxin–producing Escherichia coli
Source: Gigascience. 2019 Aug 21;8(8):giz104. doi: 10.1093/gigascience/giz104 (PMC6703438; doi:10.1093/gigascience/giz104)
Supplement: giz104_Reviewer_2_Report_Original_Submission — Oon Tek Ng -- 3/31/2019 Reviewed [file giz104_reviewer_2_report_original_submission.pdf]

Reviewer Report

Title: Comparison of single nucleotide variants identified by Illumina and Oxford Nanopore technologies in the context of a potential outbreak of Shiga Toxin Producing Escherichia coli

Version: Original Submission **Date:** 3/31/2019

Reviewer name: Oon Tek Ng

Reviewer Comments to Author:

In their paper, Greig et al. compare the performance of Oxford Nanopore Technology (ONT) and Illumina sequencing in identifying, subtyping and classifying clinical isolates in the context of outbreak investigation. This study is of considerable interest to both research and medical communities in two key aspects. Firstly, it provides an in-depth assessment of the performance of ONT versus the current sequencing standard Illumina Technology, and identifies the main mechanistic reason for discrepancies between the technologies (DNA methylation), which would be of value in optimizing and improving Nanopore analysis workflows. Secondly, they demonstrate that their real-time ONT analysis pipeline is able to rapidly provide diagnostic calls (species identification, serotyping etc) with comparable accuracy to Illumina sequencing in a fraction of the time, which has major potential applications in outbreak investigation.
Given the utility of this study, I would be happy to recommend it for publication - please consider the following points to possibly improve it further.
1) Are the methods appropriate to the aims of the study, are they well described, and are necessary controls included?
The sample size of 2 isolates is small, but justified given the context of the study (2 urgent cases of children with HUS admitted to the same hospital on the same night). However, if the authors have any other isolates sequenced (in particular, a reference isolate closely related to the reference genome) that allow for the comparison of Illumina/ONT, they could include it as supplemental information to improve the robustness of their assessment.
Run parameters for bioinformatics tools are well optimized and described. It would also be of major help to the community if the authors are willing to share the code for their real-time analysis pipeline.
The authors adequately discuss the limitations of ONT relative to Illumina sequencing with respect to their application in rapid diagnosis.
Fig 1/Methods - In the comparison of the ONT/Illumina workflows, we note that two different DNA extraction methods are used (manual + QiaSymphony cleanup for Illumina, Promega Wizard Genomic DNA Purification for ONT). Are the methods interchangeable for the purposes of the workflow?
2) Are the conclusions adequately supported by the data shown?
Analyses are generally robust and well-supported, but we would like clarification on the following points:
Line 214 - When comparing the case B ONT sequence with the 3 concurrent outbreak isolates, was it compared against Illumina sequences, or Nanopore sequences? If the comparison was between ONT and Illumina sequences, the discordance might arise from differences in the base-calling/software

methods, and might disappear if all isolates were sequenced with ONT and compared directly (or would the high error rate preclude a valid comparison?) Please clarify and comment.
Line 216-218 - Given that 7 SNPs is not too dramatic a difference one could still make the case that the cases are quite plausibly linked. Would you be able to set an approximate SNV threshold for concluding genetic linkage?
3) Please indicate the quality of language in the manuscript. Does it require a heavy editing for language and clarity?
The language of the manuscript is quite clear. Please correct "manufactures instructions" to "manufacturer's instructions".
I also feel that the title of the manuscript downplays the speed and relative accuracy of the ONT diagnostic pipeline - the focus of the title should not be on the comparison of SNVs, but rather the comparison of the overall performance of the two methods. A title reflecting this and highlighting the rapid, real-time analysis capability of ONT-based diagnostics would be able to better capture reader interest and increase the impact of the manuscript.
Similarly, the abstract should be edited to emphasize the speed and real-time analysis capability.
4) Are you able to assess all statistics in the manuscript, including the appropriateness of statistical tests used?
Yes - our group has experience analysing similar datasets. The precision/recall analysis performed is straightforward and appropriate.
(This manuscript was co-reviewed with Weizhen Xu, a postdoctoral fellow in my research group)

Methods

Are the methods appropriate to the aims of the study, are they well described, and are necessary controls included? Choose an item.

Conclusions

Are the conclusions adequately supported by the data shown? Choose an item.

Reporting Standards

Does the manuscript adhere to the journal's guidelines on minimum standards of reporting? Choose an item.

Choose an item.

Statistics

Are you able to assess all statistics in the manuscript, including the appropriateness of statistical tests used? Choose an item.

Quality of Written English

Please indicate the quality of language in the manuscript: Choose an item.

Declaration of Competing Interests

Please complete a declaration of competing interests, considering the following questions:

- Have you in the past five years received reimbursements, fees, funding, or salary from an organisation that may in any way gain or lose financially from the publication of this manuscript, either now or in the future?
- Do you hold any stocks or shares in an organisation that may in any way gain or lose financially from the publication of this manuscript, either now or in the future?
- Do you hold or are you currently applying for any patents relating to the content of the manuscript?
- Have you received reimbursements, fees, funding, or salary from an organization that holds or has applied for patents relating to the content of the manuscript?
- Do you have any other financial competing interests?
- Do you have any non-financial competing interests in relation to this paper?

If you can answer no to all of the above, write 'I declare that I have no competing interests' below. If your reply is yes to any, please give details below.

I declare that I have no competing interests.

I agree to the open peer review policy of the journal. I understand that my name will be included on my report to the authors and, if the manuscript is accepted for publication, my named report including any attachments I upload will be posted on the website along with the authors' responses. I agree for my report to be made available under an Open Access Creative Commons CC-BY license (http://creativecommons.org/licenses/by/4.0/). I understand that any comments which I do not wish to be included in my named report can be included as confidential comments to the editors, which will not be published.

I agree to the open peer review policy of the journal

To further support our reviewers, we have joined with Publons, where you can gain additional credit to further highlight your hard work (see: https://publons.com/journal/530/gigascience). On publication of this paper, your review will be automatically added to Publons, you can then choose whether or not to claim your Publons credit. I understand this statement.

Yes Choose an item.
