# Supplementary material for: Comparison of single-nucleotide variants identified by Illumina and Oxford Nanopore technologies in the context of a potential outbreak of Shiga toxin–producing Escherichia coli
Source: Gigascience. 2019 Aug 21;8(8):giz104. doi: 10.1093/gigascience/giz104 (PMC6703438; doi:10.1093/gigascience/giz104)
Supplement: giz104_Reviewer_3_Report_Original_Submission — Brian Forde -- 4/2/2019 Reviewed [file giz104_reviewer_3_report_original_submission.pdf]

Reviewer Report

Title: Comparison of single nucleotide variants identified by Illumina and Oxford Nanopore technologies in the context of a potential outbreak of Shiga Toxin Producing Escherichia coli

Version: Original Submission **Date:** 4/2/2019

Reviewer name: Brian Forde

Reviewer Comments to Author:

The authors examine the feasibility of using Nanopore sequencing to characterise single nucleotide variants in clinically relevant outbreaks. The authors present an interesting and relevant comparison of sequencing technologies given the rapid uptake of WGS as a clinical diagnostic tool.The data set is clearly described and accession code for all data submitted to the SRA are available in the manuscript and online. The methods are well described and all software/Bioinformatic tools are available online. Although it can be addressed, my main concern with the manuscript is that the research was part funded by Oxford Nanopore. I believe the authors have not fully addressed the limitations of the Nanopore sequencing technology e.g. Cost, variability in throughput, etc. I? have highlighted some of these issues below. Platform limitations will also need to be included in the discussion
In the abstract and results the authors make a comparison of Illumina and ONT workflows of the time taken from DNA extraction to availability of results. The authors state that typing data (Shiga toxin subtyping and serotyping) was available within 7 hours while with Illumina it took ~40 hours to get these results. How do these time frames compare to standard laboratory based typing techniques that might be available in a diagnostic/pathology lab?
I would like to see a comparison of the sequencing costs for Illumina and Nanopore sequencing. A comparison against standard laboratory based typing techniques might also be beneficial to a broader audience (i leave that to the authors to decide).
The authors state that the genetic relatedness of isolates could be determined at ~6 hours. I assume by genetic relatedness we are talking about variant/SNP typing. Can the authors explain how variants could be determined at 6 hours yet a MLST profile for Case B could not be determined until 10 hours had passed? Surely an inability to determine a MLST type indicates that the genome has not yet been sufficiently covered and it therefore unsuitable for variant typing.
Additionally, in order to be cost effective multiple samples would need to sequenced on the same flowcell at the same time. Can the authors comment on what impacted multiplexing might have on the time frames described here?
Can the authors comment as to why a number of regions (all describes as prophage with the exception of 1 in case A) were only present in the ONT-only chromosome assemblies?
I find it odd that these regions were not present in the Illumina sequence data and are also absent from the hybrid assembly. can the authors comment on why this might be the case and what impact that might have for genome sequencing and assembly strategies moving forward?
With regards to the genome assemblies of case A and Case B can the authors provide information on the number of erroneous indels that were present in the Nanopore assemblies?

I assume these errors were polished out but did the authors only use Nanopore sequence data or was Illumina data also required.?
Line 129: Remove the MLST allele numbers
Line 385: form -> for(?)
Table 1 is very hard to interrupt. Consider restructuring the table.

Methods

Are the methods appropriate to the aims of the study, are they well described, and are necessary controls included? Choose an item.

Conclusions

Are the conclusions adequately supported by the data shown? Choose an item.

Reporting Standards

Does the manuscript adhere to the journal's guidelines on minimum standards of reporting? Choose an item.

Choose an item.

Statistics

Are you able to assess all statistics in the manuscript, including the appropriateness of statistical tests used? Choose an item.

Quality of Written English

Please indicate the quality of language in the manuscript: Choose an item.

Declaration of Competing Interests

Please complete a declaration of competing interests, considering the following questions:

- Have you in the past five years received reimbursements, fees, funding, or salary from an organisation that may in any way gain or lose financially from the publication of this manuscript, either now or in the future?
- Do you hold any stocks or shares in an organisation that may in any way gain or lose financially from the publication of this manuscript, either now or in the future?
- Do you hold or are you currently applying for any patents relating to the content of the manuscript?
- Have you received reimbursements, fees, funding, or salary from an organization that holds or has applied for patents relating to the content of the manuscript?
- Do you have any other financial competing interests?

- Do you have any non-financial competing interests in relation to this paper?

If you can answer no to all of the above, write 'I declare that I have no competing interests' below. If your reply is yes to any, please give details below.

No Competing interest

I agree to the open peer review policy of the journal. I understand that my name will be included on my report to the authors and, if the manuscript is accepted for publication, my named report including any attachments I upload will be posted on the website along with the authors' responses. I agree for my report to be made available under an Open Access Creative Commons CC-BY license (http://creativecommons.org/licenses/by/4.0/). I understand that any comments which I do not wish to be included in my named report can be included as confidential comments to the editors, which will not be published.

I agree to the open peer review policy of the journal

To further support our reviewers, we have joined with Publons, where you can gain additional credit to further highlight your hard work (see: https://publons.com/journal/530/gigascience). On publication of this paper, your review will be automatically added to Publons, you can then choose whether or not to claim your Publons credit. I understand this statement.

Yes Choose an item.
